# Does network topology influence systemic risk contribution? A perspective from the industry indices in Chinese stock market

**DOI:** 10.1371/journal.pone.0180382

**Published:** 2017-07-06

**Authors:** Haiming Long, Ji Zhang, Nengyu Tang

**Affiliations:** 1 College of Finance and Statistics, Hunan University, Changsha, China; 2 Lally School of Management, Rensselaer Polytechnic Institute, New York, United States of America; Universidad Rey Juan Carlos, SPAIN

## Abstract

This study considers the effect of an industry’s network topology on its systemic risk contribution to the stock market using data from the CSI 300 two-tier industry indices from the Chinese stock market. We first measure industry’s conditional-value-at-risk (CoVaR) and the systemic risk contribution (*ΔCoVaR*) using the fitted time-varying t-copula function. The network of the stock industry is established based on dynamic conditional correlations with the minimum spanning tree. Then, we investigate the connection characteristics and topology of the network. Finally, we utilize seemingly unrelated regression estimation (SUR) of panel data to analyze the relationship between network topology of the stock industry and the industry’s systemic risk contribution. The results show that the systemic risk contribution of small-scale industries such as real estate, food and beverage, software services, and durable goods and clothing, is higher than that of large-scale industries, such as banking, insurance and energy. Industries with large betweenness centrality, closeness centrality, and clustering coefficient and small node occupancy layer are associated with greater systemic risk contribution. In addition, further analysis using a threshold model confirms that the results are robust.

## Introduction and literature review

After the financial crisis in 2008, the stability of the financial system has gradually become the focus of the industry, academia and regulators. Kaufman and Scott [1] define systemic risk as "the probability of collapse of individual or component of a group resulting in the risk of an overall system or the probability of loss of the system, and the collapse of an overall system arises from the domino effect of a single individual which has suffered the risk." The International Monetary Fund, the Financial Stability Board and the Bank for International Settlements (BIS) [2] state that systemic risk refers to a type of risk that may damage the financial system partially or totally and create a wide range of disturbances in financial services and have serious impact on the real economy.

The accurate measurement of a system’s financial risk and the identification of components that affect the entire financial system have played an important roles in the identification of systemic risk. Therefore, the academic community is mainly focused on the measurement of systemic risk. Bisias et al. [3] documented a total of six categories, including more than thirty types of systemic risk measurement, among which the most common type is the cross-sectional method. Acharya et al. [4] developed two new systemic risk measurement methods, systemic risk loss (SES) and marginal expectation loss (MES), based on expected shortfall. On this basis, Brownlees and Engle [5] proposed the SRISK index and developed the DCC-GARCH model to estimate the marginal expected loss. Adrian and Brunnermeier [6] proposed a CoVaR method based on the VaR method; they also defined CoVaR and estimated it using quantile regression. CoVaR is a strong operational method that is used to study the risk situation of financial institutions or financial markets when they suffer financial risks. The author found that CoVaR was related to the company's leverage, maturities and asset size. Lopez-Espinosa et al. [7] used the CoVaR approach to identify the main factors behind systemic risk in a set of large international banks. They found that short-term wholesale funding is a key determinant in triggering systemic risk episodes. Reboredo et al. [8] studied systemic risk in European sovereign debt markets before and after the onset of the Greek debt crisis; they took CoVaR, characterized and computed using copulas, as a systemic risk measure. Huang et al. [9] studied the systemic risk of a group of major financial institutions and proposed a measure of systemic risk based on the price of insurance against financial distress. This indicator is determined by the bank's default probability, asset relevance and macroeconomic variables.

Financial markets are a typical complex systems. The major players of the financial system perform risk transmission through their financial correlations. Network theory is of interest to various components of the financial world, e.g., description of the systemic structure and analysis and evaluation of penetration or contagion effects [10]. Therefore, many scholars have studied financial networks and risk transmission by concentrating on the interbank network and empirical research of risk transmission. These networks, which include interbank borrowing, payment networks, counterparty exposure in credit default swaps, and trade credits between companies, employ empirical and simulation techniques to assess the propagation of institution failures [11–15]. The empirical study of Allen and Gale [16] showed that a fully structured network is more stable than an incomplete structural network. Lubloy [17] found that the structure of the interbank network has a significant impact on the spread of bank defaults in the Hungarian banking industry. Empirical research has shown that the node degree of the bank network exhibits a power-law distribution for the US FedWire system, the Austrian interbank market, the Brazilian banking system, the UK and the Italian market [15,18,19]. The size of the initial collapsed bank is the dominant factor influencing transmission, but the characteristics of the interbank network are the most important factors affecting the extent of risk propagation [20]. However, the literature on network analysis only concentrates on the overall network structure’s effect on systemic risk; the relationship between the local network structure and systemic risk contribution is neglected [21]. Many scholars have evaluated the systemic risk by using the cross-correlation between financial institutions to build a financial network to study the basic topology characteristics and the inherent hierarchical structure of the network [22–28]. The results illustrate that the interconnectedness of stock return correlation networks has increased and correlation networks more accurately represent systemic risk than do physical networks. Moreover, the structure of correlation networks is vulnerable to macroeconomic shocks. Huang et al. [29] studied the effects of financial institutions’ topology in the financial network on their systemic risk contribution and found that they were correlated. However, the measurement of systemic risk and the construction of the stock network in the above literature are conducted from the viewpoint of financial institutions. There are also many studies on the evolution of dynamic correlations among industry indices from the perspective of the industries in the stock market. A common finding is that the topology of the industry index correlation network changes during a crisis [30, 31]; therefore, it is natural to investigate systemic risk from the perspective of the industry index network in the stock market. Uechi et al. [32] presented a new measure (SDR) to quantify the evolution of the economic sector’s activity reflected in financial markets. They found that an increase in SDR indicated increased in systemic risk. Raddant and Kenett [33] analyzed the dependencies among nearly 4000 stocks from15 countries and found that the energy, raw materials and financial sectors played important roles in connecting markets and that changes in the energy and raw materials markets affected the correlations among stock industries.

However, few studies have concentrated on measuring systemic risk contribution from an industry’s perspective, or on establishment of an industry index network. Researchers have failed to explore the relationship among systemic risk contribution in industry and the local structure of the industry’s network. On one hand, the flow of information can effectively explain the linkage effect of a stock [34], and information that affects stock market volatility includes common information and private information [35]. The emergence of common information including macroeconomic fundamentals, can lead to simultaneous fluctuations in the stock indices of different industries. When private information appears, cross-industry asset transfer behavior also contributes to correlation among industries. On the other hand, there are associations between different industries in the economy, which are reflected by the industry index correlation effect [33]. The impact of any industry is transmitted through the index correlation between industries. Therefore, it is necessary to measure the contribution to systemic risk’s from the perspective of the stock industry and to establish an industry index network to explore the relationship between the industry’s contribution to systemic risk and the local network structure of the industry.

When constructing networks, in much of the literature, the vertex of the stock network is the stock, and the edge between the vertexes is the correlation of the stock price [36–42]. A complete stock network, which contains redundant information is usually very large; thus, special filtering methods are used to reduce complexity, such as threshold, minimum spanning tree (MST) [38–41] and plane maximum filter graph (PMFG) [43]. Kenett [44] introduced a partial correlation network to detect the prominent role of financial stocks in controlling the correlation structure of the market. To understand the functions of a network, one must study its dynamic properties [10]. The rolling correlation coefficient (RC) method is used to obtain the correlation coefficient matrix [45–47] in many studies analyzing dynamic networks. However, on one hand, using the rolling window technique to construct stock networks may produce different results due to researchers’ specific selection of parameters, such as the length and drift of the estimation window, which undermines the objectivity and reasonability of the research conclusions [26]. On the other hand, due to the volatility of stock market linkage, the estimations produced from the data may have errors due to heteroscedasticity and variance clustering. Therefore, RC is generally not considered suitable for high-frequency financial data. Many scholars employ other methods to estimate the dynamic correlation coefficient, such as Trancoso [48], who used the dynamic correlation estimated by BEKK model to construct a dynamic global economic network. Lyocsa et al. [49] who estimated the dynamic conditional correlation using DCC MV-GARCH model [50,51];and Wang et al. [52], who studied the dynamic correlation structure of a foreign exchange market by adopting a time-varying copula and MST method. The empirical results show that the market dynamics are better preserved by DCC.

The current research on stock market networks is concentrated on developed economies. Research on the stock market networks of emerging economies is rare and mainly focuses on interindustry dependencies rather than the dependent relationships of financial institutions. Thus, the gap in the existing literature can be filled by studying the relationship between the network topology and the contribution of systemic risk from the perspective of the stock industry in China. This study measures CoVaR and *ΔCoVaR* using the time-varying t-copula function using data from the CSI 300 two-tier industry indices on the Chinese stock market. Then, the stock industry network and its MSTs are constructed using the DCC. We then investigate the nature of the connection and the topology of the network by estimating the local network structure of each industry, including the node strength, betweenness centrality, closeness centrality, node occupation layer and clustering coefficient. Finally, we analyze the relationship between the network topology and the industry’s systemic risk contribution to the stock industry using seemingly unrelated regression estimation (SUR) of panel data.

The remainder of this paper is organized as follows. Section 2 discusses the applied methodology; the main empirical results and analysis are presented in Section 3. Finally, Section 4 summarizes and concludes the paper.

## Model introduction

### CoVaR measurement

VaR is the dominant method in traditional measurments of market risk. VaR represents the maximum possible loss of an asset or portfolio at a particular time in the future based on a certain probability level, and it is satisfied by:

$$Pr(x_{i}\geq VaR_{q}^{i})=1\;\text{-}\;q$$ (1)

Adrian and Brunnermeier [6] established a risk-taking model on the basis of the VaR model called CoVaR, which considers the relationship of risk spillovers between financial institutions and financial sectors. CoVaR represents the maximum possible loss of other assets or portfolios when a certain asset is equal to VaR at a future time at a certain probability level. Thus, when the loss value of individual i is VaR, the CoVaR of j at confidence level 1-q is as follows:

$$Pr(X_{i}\leq CoVaR_{q}^{j/i}/X^{i}=VaR_{q}^{i})=q$$ (2)

In contrast with the unconditional value $VaR_{q}^{j}$, CoVaR considers the spillover and transmission of financial market risk and can reflect an increase in individual relevance during a crisis, which is an improvement over the traditional VaR model. Similarly, the marginal contribution of component i to systemic risk is the difference between the conditional risk and the unconditional risk of the financial system when the maximum possible loss is incurred by component i. This marginal contribution can be expressed as follows:

$$\Delta CoVaR_{s/i}^{t}=CoVaR_{q}^{s/L_{i}=VaR_{q}^{i}}-CoVaR_{q}^{s/L_{i}=VaR_{50\%}^{i}}$$ (3)

### CoVaR estimation

As a marginal distribution of each index return, the specific form of the model is given in formulas (4)-(6), where *t*_*d*_ is the t distribution with degrees of freedom d.

$$R_{t}=\mu +fR_{t-1}+\varepsilon_{t}\sigma_{t}$$ (4)

$$\sqrt{\frac{d}{d-2}}g\varepsilon_{t}∼i.idt_{d}$$ (5)

$$\sigma_{t}^{2}=\omega +A\varepsilon_{t-1}^{2}\sigma_{t-1}^{2}+B\sigma_{t-1}^{2}$$ (6)

According to Roncalli [53], the t-copula function $C_{\rho ,d}^{t}(u,v)$ can be expressed as follows:

$$C_{\rho ,d}^{t}(u,v)=\int_{0}^{u}t_{d+1}\left({\left(\frac{d+1}{d+{\left[t_{d}^{\text{-}1}(u)\right]}^{2}}\right)}^{\frac{1}{2}}\frac{t_{d}^{\text{-}1}(d)\text{-}\rho t_{d}^{\text{-}1}(t)}{\sqrt{1\text{-}\rho^{2}}}\right)dt$$ (7)

According to Hawka [54], $CoVaR_{\alpha }^{s}$ can be expressed as follows:

$$g(v,u)=\frac{\partial C_{\rho ,d}^{t}(u,v)}{\partial u}=t_{d+1}\left({\left(\frac{d+1}{d+{\left[t_{d}^{\text{-}1}(u)\right]}^{2}}\right)}^{\frac{1}{2}}\frac{t_{d}^{\text{-}1}(d)\text{-}\rho t_{d}^{\text{-}1}(t)}{\sqrt{1\text{-}\rho^{2}}}\right)$$ (8)

$$CoVaR_{\alpha }^{s}=F_{s}^{\text{-}1}\left(t_{d}\left(\rho t_{d}^{\text{-}1}(\beta )+\sqrt{\frac{\left(1\text{-}\rho^{2})(d+{\left[t_{d}^{\text{-}1}(\beta )\right]}^{2}\right)}{d+1}t_{d+1}^{\text{-}1}(\alpha )}\right)\right)$$ (9)

where *F*_*s*_ is the marginal distribution of system losses, *ρ*_*t*_ represents the dynamic correlation coefficient, which evolves through time as in the DCC(1,1)model of Engle [51], and *ρ*_*t*_ satisfies the following equation:

$$Q_{t}=(1\text{-}\alpha \text{-}\beta )g\bar{Q}+\alpha \varepsilon_{t\text{-}1}g\varepsilon_{t\text{-}1}^{\prime }+\beta gQ_{t\text{-}1}$$ (10)

$$\rho_{t}={\tilde{Q}}_{t}^{\text{-}1}Q_{t}{\tilde{Q}}_{t}^{\text{-}1}$$ (11)

where *ρ*_*t*_ is the DCC coefficient and *α* and *β* are non-negative constants that satisfy *α* + *β* < 1. $Q_{t}=\left[\begin{array}{cc} q_{t}^{ii} & q_{t}^{ij}\\ q_{t}^{ji} & q_{t}^{jj} \end{array}\right]$, ${\tilde{Q}}_{t}=\left[\begin{array}{cc} 0 & \sqrt{q_{t}^{ij}}\\ \sqrt{q_{t}^{ji}} & 0 \end{array}\right]$ is the conditional standard deviation matrix, $\bar{Q}$ is the residual unconditional variance matrix, and $q_{t}^{ij}$ is the covariance between variables $R_{t}^{i}$ and $R_{t}^{j}$ at time t.

### Construction of the dynamic network

This paper takes the industry index as the network node, and various industry nodes are connected by the weighted edge of the return rate of the dynamic correlation coefficients. We use the DCC method to compute all t period bivariate DCCs and obtain the N × N correlation matrix CDCCt. After performing these procedures, we transform the correlation coefficients to distance metrics between each pair of CSI 300 two-tier industry indices as in Mategna [55], i.e., $d_{i.j}^{t}=\sqrt{2(1\text{-}\rho_{i.j}^{t}})$, (-*1 ≤ ρ*_*t*_ *≤ 1*, $0\leq d_{i.j}^{t}\leq 2$). Then, the N×N distance matrix is formed. Because there is substantial noise in the initial correlated network, only some edges contain useful and relevant information. Therefore, it is necessary to filter out the excess edges from the initial connected network to determine the true sense of the network. We utilize Kruskal’s algorithm to filter the network information and to construct the minimum spanning tree (*MST*^*t*^) corresponding to *G*^*t*^.

### Network topology

#### Node strength

The node strength is defined as the sum of the correlation coefficients of the node i with all other nodes to which it is linked; for example, where the node strength of node i is defined as:

$$S_{i}^{t}=\sum \rho_{i.j}^{t}$$ (12)

The node strength represent the effect of a node on the stock price of other nodes in the network. The greater node i’s strength is, the greater the node’s impact on other nodes in the network.

#### Node betweenness centrality

Node betweenness centrality is defined as the number of geodesics (shortest paths) going through a vertex and is a measure of one node’s importance as an intermediate element between other nodes in the network. Node betweenness centrality is calculated using the following formula:

$$B_{i}^{t}=\sum_{j<k}g_{j,k}^{i}/g_{j,k}$$ (13)

where $g_{j,k}^{i}$ represents the number of shortest geodesic paths traversing i between nodes j and k, *g*_*j*,*k*_ represents the number of short paths between the j and k. An industry with high betweenness centrality would have an important effect on other industries since it can stop or distort the information that passes through i

#### Node closeness centrality

Closeness centrality represents the sum of the distances between a node and all other nodes in the graph. The closeness centrality of node i is defined as:

$$F_{i}^{t}=\sum_{j\in G.j\neq i}l_{i.j}$$ (14)

where *I*_*i*.*j*_ is the shortest distance between industry i and j. Closeness centrality is an indicator of the importance of nodes to the entire network, and an industry with low closeness centrality is less dependent on other intermediary industries to receive messages. In our work, this measure is related to the industry’s capacity to propagate transmission.

#### Node occupation layer

C is the node with the largest degree in the smallest spanning tree. The node occupancy layer of point i, the minimum distance from i to c, is defined as $l_{i,c}^{t}$. The node occupancy layer of c is 0, which reflects the extent of closeness to the central position in the network. The farther from the central position an industry index is, the weaker it is in information resources, power, prestige and influence.

#### Node clustering coefficient

The original network *G*^*t*^ with edge weights, equal to the DCC can be used to calculate the node clustering coefficient. The clustering coefficient of node i is defined as:

$$C_{i}^{t}=\frac{1}{N(N\text{-}1)}\sum_{j,k}(\left|\rho_{i,j}^{t}\right|\times \left|\rho_{i,k}^{t}\right|\times \left|\rho_{j,k}^{t}\right|{)}^{\frac{1}{3}},i\neq j,i\neq k,j\neq k.$$ (15)

## Empirical research

### Selected data

The CSI 300 index and its two-tier index were selected as the research sample. CSI 300 represents the stock market system, and the CSI 300 two-tier industry indices, including 17 industries, such as banks, comprehensive finance and capital goods, represent the components of the stock market system. The CSI 300 index, which was jointly issued by the Shanghai and Shenzhen Stock Exchanges on April 8, 2005, is compiled from a sample of 300 A shares from the Shanghai and Shenzhen stock markets. The CSI index covers approximately 60% of the market value of the Shanghai and Shenzhen markets, with good market representation. The following experimental data were acquired from WIND information database for the period between January 1, 2007 and September 30, 2016 (2372 consecutive trading days in total): daily closing prices of the CSI 300 index and its two-tier industry indices, quarterly financial data of the companies included in the CSI 300 secondary industry index, GDP growth rate, CPI growth rate, interbank overnight rate, dollar against the RMB exchange rate and other macroeconomic data. The list of two-tier industries and their codes and respective symbols are presented in Table in S1 Table.

### DCC-copula estimation

We first calculate the DCCs between the 17 industry indices returns, and the DCCs between the returns of the industry indices and the CSI 300 index, resulting in 153 pairs of return sequences. The DCCs are calculated as follows: (1) The running unit root test, Ljung-Box autocorrelation test and ARCH effect test is performed for each industry index return and the CSI 300 index return. The results are shown in Table 1. (2) According to the fitting effect, the AR (m) -GARCH (p, q) -t model is used to fit the marginal distribution of each return and can be used to calculate the standard residual sequence. (3) The time-varying copula function is used to fit the standard residual sequence of each pair of returns, and the DCCs are calculated.

**Table 1:** ADF, Ljung-Box and ARCH effect test results for the ENG (CSI 300 Energy index) and the CSI 300 index returns.

	ADF	Q(5)	Q(10)	Q^2^(5)	Q^2^(10)
**ENG**	-47.5403	42.207	51.857	289.429	506.656
	(0.000)	(0.000)	(0.000)	(0.000)	(0.000)
**CSI 300**	-47.308	18.782	32.517	69.799	79.906
	(0.000)	(0.002)	(0.000)	(0.000)	(0.000)

Due to limited space, a list of the DCCs between the ENG and the CSI 300 index return sequence is given as an example to illustrate the overall measurement process. Table 1 presents the results of the ADF, Ljung-Box and ARCH effect test on the ENG and CSI 300 index return sequence. The small p-value indicates that the index returns are stable. The p-value of Ljung-Box test of the two series returns is less than 0.05; therefore, the null hypothesis is rejected at the 5% significance level. The p-values of the ARCH effect of the two series of return sequences are very small. The data shows heteroscedasticity with a significant ARCH effect. Therefore, we adopt the ARMA and GARCH models to perform the estimation; the estimates for the ENG are as follows:

$$r_{i}^{t}=0.0506+0.0117r_{i}^{t\text{-}1}+\varepsilon_{i}^{t}$$

$${\left(\sigma_{i}^{t}\right)}^{2}=0.0169+0.0558\;{\left(\varepsilon_{i}^{t\text{-}1}\right)}^{2}+0.9422\;{\left(\sigma_{i}^{t\text{-}1}\right)}^{2}$$

We performed an autocorrelation test and ARCH effect test for the residual sequence to ensure the validity of this model to describe the heteroscedasticity and autocorrelation of the ENG. The t-value of the autocorrelation test of ENG is 12.3493, and the p-value is 0.0771, indicating that the sequence has no autocorrelation. The p-value of the ARCH effect test of 0.9991 indicates that the ARCH effect does not exist. Therefore, the AR-GARCH model can accurately describe the autocorrelation and conditional heteroscedasticity of the original return sequence. The GARCH regression results of the CSI 300 index are as follows:

$$r_{i}^{t}=\text{-}0.0242\text{-}0.0082r_{i}^{t\text{-}1}+\varepsilon_{i}^{t}$$

$${\left(\sigma_{i}^{t}\right)}^{2}=0.0192+0.0709\;{\left(\varepsilon_{i}^{t\text{-}1}\right)}^{2}+0.9291\;{\left(\sigma_{i}^{t\text{-}1}\right)}^{2}$$

**Fig 1:**
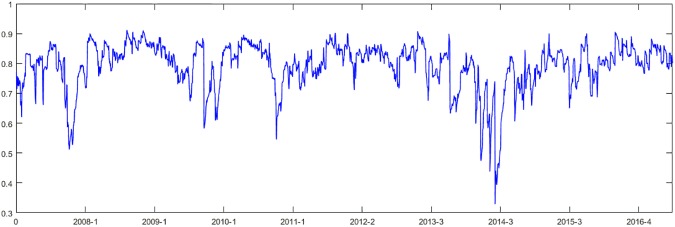
The DCCs between the ENG and CSI 300 index returns from January 2007 to June 2016.

The results of the autocorrelation and ARCH effect tests show that the model fit is good. Then, we use the standard deviation DDC model to obtain *α* = 0.04805 and *β* = 0.9157. As shown in Fig 1, the maximum DCC between the ENG and CSI 300 index is 0.9115, the minimum is 0.3287, the mean is 0.7922, and the variance is 0.0783. The skewness and kurtosis are -1.7230 and 7.2698, respectively, representing the characteristics of the peak and the thick tail of graph. We then calculate the correlation coefficient of the autocorrelation function (ACF); from 1 to 20 days, ACF is positive with a 1 to 20 day lag at the 95% confidence level. In the autocorrelation test, Q (5) = 9229.3, Q (10) = 15434, and the p-value is zero; thus, the DCC has significant positive autocorrelation.

### CoVaR estimation

**Fig 2:**
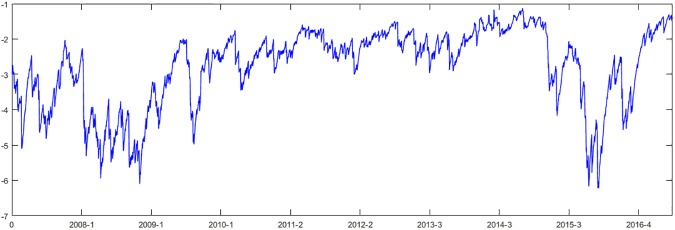
The systemic risk contribution of ENG from 2007 to 2016.

In this section, the DCCs between the CSI 300 index and CSI two-tier industry indices are utilized to measure the systemic risk contributions of the industries. The ENG is taken as an example, and the evaluation of time-varying systemic risk contribution at the 95% confidence level is shown in Fig 2. A smaller *ΔCoVaR* indicates larger systemic risk contribution of the industry. The maximum value is -1.1226, and the minimum value is -6.2235.

The value and standard deviation are -2.7782 and 1.0978, respectively. The standard deviation of the systemic risk contribution in each year from 2007 to 2016 is 0.6722, 0.2718, 0.9797, 0.3553, 0.2824, 0.3225, 0.3490, 0.4711, 1.1219, and 1.0015. Due to the financial crisis, *ΔCoVaR* fluctuates drastically from 2007 to 2009, and the volatility of *ΔCoVaR* has since decreased, with an upward trend from 2010 to 2014. Furthermore, due to the stock market crash, *ΔCoVaR* has fluctuated more drastically in 2015 and 2016. We also estimate the ACF of the correlation coefficient, and the results show that the ACF value is positive, with 1 to 40 days lag at the 5% level. The autocorrelation test gives Q (5) = 11108.3171, Q (10) = 21107.2639, indicating significant positive autocorrelation and that the systemic risk contribution of the ENG is stable and predictable. The systemic risk contribution of the ENG is increasing. The average *ΔCoVaR* of each CSI 300 two-tier industry index is ranked in ascending order in Table 2. The smaller an industry’s *ΔCoVaR* is, the greater its systemic risk contribution. The top three industries are CRE, FBA, and DAP, and the bottom three are CBI, INS and ENG. The systemic risk contribution difference between CRE, which ranks first, and ENG, which ranks last, is -1.7988.

**Table 2:** Industry ranking based on systemic risk contributions.

Ranking	Code	Industry	*ΔCoVaR*
**1**	CRE	300 Real Estate	-4.5771
**2**	FBA	300 Food&Beverage	-4.1272
**3**	DAP	300 Durables&Apparel	-3.8601
**4**	ITF	300 IT Software	-3.6565
**5**	UTL	300 Utilities	-3.5450
**6**	ITH	300 IT Hardware	-3.5032
**7**	CAG	300 Capital Goods	-3.4832
**8**	RET	300 Retail	-3.4324
**9**	CTR	300 Trans	-3.4126
**10**	MEA	300 Media	-3.3974
**11**	CMA	300 Materials	-3.2336
**12**	ACP	300 Auto&Component	-3.1751
**13**	DFI	300 Diversified financials	-3.1250
**14**	PBT	300 Pharma& Biotech	-2.8640
**15**	CBI	300 Banks	-2.8293
**16**	INS	300 Insurance	-2.7964
**17**	ENG	300 Energy	-2.7782

### Network construction and network topology

**Fig 3:**
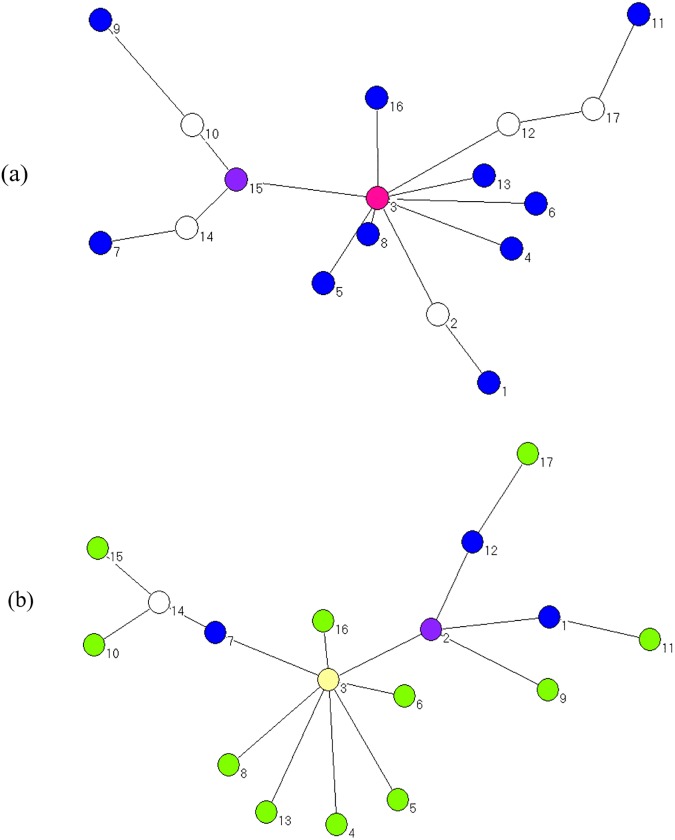
MSTs of the CSI 300 two-tier industry index network on January 4, 2007 (a) and September 30, 2016(b).

**Fig 4:**
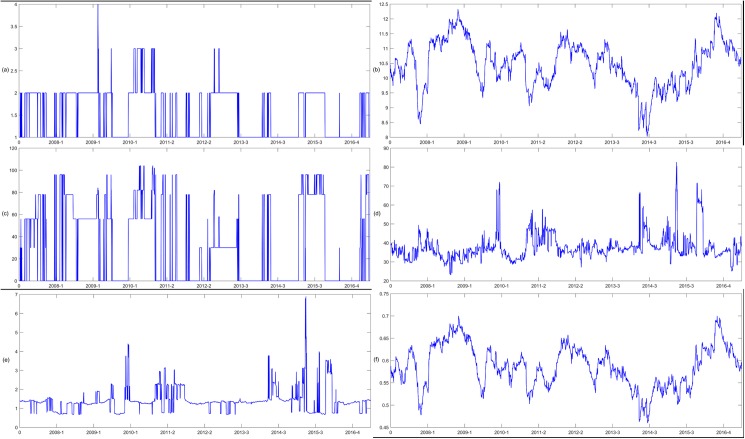
The evolution of the topology of the ENG. (a) shows the node degree of the ENG. (b) shows the node strength of the ENG. (c) shows the betweenness centrality of the ENG. (d) shows the closeness centrality of the ENG. (e) shows the node occupation layer of the ENG. (f) shows the clustering coefficient of the ENG.

After calculating all the bivariate DCCs, we construct the network *G*^*t*^ and the corresponding *MST*^*t*^ in period t. The MST of the first and last days of the sample period are shown in Fig 3, which shows a significant change in the relative position of the node in the two networks. Thus, the network structure has also changed during this period. For example, at t = 1, the node with the largest degree is CAG, and the top three nodes are CAG, ITH, and DFI. At t = 2372, the top three nodes are CAG, ITF and CMA. The evolution of the topology of the ENG in the dynamic network changes is shown in Fig 4. The ENG’s node degree ranges from0 to 4, its node strength ranges from 6.4063 to 13.4470, its node betweenness centrality ranges from 0 to 228, its node closeness centrality ranges from 15.4338 to 83.8775, its node occupation layer ranges from 0 to 7.1972 and node clustering coefficient ranges from 0.3860 to 0.7492.

### The effect of network topology on system risk contribution

A change in the network topology of the stock industry indicates that the information transmission relationship between industries and the industry association and among economic industries may also change, which may lead to a change in the systemic risk contribution of the industry to the stock market. In this section, we investigate the effects of network topology on systemic risk using SUR of the panel data. Previous studies have shown that asset size, leverage, and return on assets have substantial impact on systemic risk, therefore, the logarithms of the industry assets, leverage, and asset returns are used as the additional control variables. The data are obtained from sub-sector quarterly financial reports, semi-annual reports and annual reports. The financial data of each industry stock are aggregated to obtain the industries' overall financial data and the required agent index data. Since macroeconomic activity affects the systemic risk contributions of the industries in the stock market and the stock industry network structure, it is included to control the main impact of the following economic activities: GDP growth rate, CPI growth rate, bank overnight lending rate, and US dollar to RMB exchange rate. We convert the quarterly financial data, quarterly GDP growth rate data and monthly CPI growth rate data, into daily data to match the timescale of other variables.

We initially conduct stationary tests on these variables. The results in Table 3 indicate that the variables are stationary variables. Then, the Hausman test is applied to determine whether the model has random effects or fixed effects. The null hypothesis is that the random effect model should be established as follows:

$$\Delta CoVaR_{s/i}^{t}=\alpha +x_{i}^{t}\beta +v_{i}+u_{i}^{t}$$

where $x_{i}^{t}=(S_{i}^{t},B_{i}^{t},F_{i}^{t},l_{i}^{t},C_{i}^{t},lnAssets_{i}^{t},leverage_{i}^{t},ROA_{i}^{t},GDPgr^{t},CPIgr^{t},R^{t},Ex^{t})$, *α* is a constant part of the intercept term, *v*_*i*_ is a random part of the intercept term, *β* is the12×1 coefficient matrix, i = 1,2,…,N, and t = 1,2,…,T. The Hausman test statistic is 19.80, with a p-value of 0.01; the null hypothesis is rejected and a fixed effects model is established. Next, constant coefficient, variable intercept and variable coefficient tests are conducted. The sum of residual squares in the variable coefficient model, variable intercept model and fixed parameter model is S1 = 39,465.4883, S2 = 55,695.4629, S3 = 64,338.4884. According to Cheng [56], since N = 17, T = 2372, and k = 12, *F*_*1*_ = 79.2892 and *F*_*2*_ = 121.5134 at 95% confidence level, with *F*_*1α*_ = 1.1741 and *F*_*2α*_ = 1.1671. *F*_*1*_ and *F*_*2*_ are larger than their corresponding critical values; therefore, the variable coefficient model is chosen. The model is shown below:

$$\Delta CoVaR_{s/i}^{t}=\alpha_{i}+x_{i}\beta_{i}+u_{i}$$

where i = 1,2,…,N, $\Delta CoVaR_{s/i}^{t}$ is a T × 1 vector of independent variables, *x*_*i*_ is the *T* × *k* explanatory variable matrix, *α*_*i*_ is the intercept term, which varies by industry, *β*_*i*_ is the *k* × *1* coefficient matrix, which varies by industry, and *u*_*i*_ is the residual matrix. The Breusch-Pagan LM statistic for the variable coefficient model is 78,263.684 with a p value of 0, and the null hypothesis, which is not relevant to the same period, can be rejected. Therefore, SUR is adopted, and the regression results are shown in Table 4.

**Table 3:** Stationary test results.

	CoVaR	S	B	F	l	C	lnAsset
**ADF**	267.38	150.18	1178.23	1162.05	1225.48	132.14	321.75
	(0.000)	(0.000)	(0.000)	(0.000)	(0.000)	(0.000)	(0.000)
	**leverage**	**ROA**	**GDPgr**	**CPIgr**	**R**	**Ex**	
**ADF**	212.38	56.69	56.50	136.54	56.69	274.46	
	(0.000)	(0.009)	(0.009)	(0.000)	(0.009)	(0.000)	

Note: S represents node strength. B represents betweenness centrality network center. F represents the closeness centrality of network. l represents node occupancy layer of network, C represents cluster coefficient of network. lnAsset represents logarithm of asset. Leverage represents assets and liabilities. ROA represents return on assets. GDPgr represents GDP growth rate. CPIgr represents CPI growth rate. R represents bank overnight lending rate. Ex represents US dollar to RMB exchange rate.

**Table 4:** Panel data model estimation results.

Industry	S	B	F	l	C	lnAsset	leverage	ROA	Common factors
**CRE**	1.2183^***^	-0.0018^***^	-0.0209^***^	0.1727^***^	-33.5335^***^	0.4874^***^	-1.1255^***^	-0.7924^***^	Controlled
	(20.28)	(-7.38)	(-10.32)	(9.07)	(-28.02)	(8.99)	(-15.68)	(-2.84)	
**FBA**	-2.1005^***^	-0.0008^*^	-0.1225^***^	0.5526^***^	-22.3702^***^	0.3185	1.5521	-25.6004^***^	Controlled
	(-8.37)	(-1.74)	(-17.14)	(7.83)	(-5.63)	(1.02)	(1.63)	(-15.33)	
**DAP**	1.0075^***^	0.0019^***^	-0.0205^***^	0.0526	-34.9328^***^	0.2552^***^	-1.6503^***^	-9.5159^***^	Controlled
	(8.53)	(4.01)	(-4.56)	(1.47)	(-18.82)	(7.28)	(-5.12)	(-8.98)	
**ITF**	1.0394^***^	-0.0023^***^	-0.0531^***^	0.3378^***^	-33.8434^***^	0.4193^***^	-3.5190^***^	-13.1750^***^	Controlled
	(7.65)	(-6.43)	(-17.23)	(8.21)	(-14.45)	(3.84)	(-11.81)	(-15.55)	
**UTL**	-1.6424^***^	-0.0077^***^	-0.0434^***^	0.2105^***^	11.1274^***^	0.5415^***^	0.7533^***^	2.1188^***^	Controlled
	(-13.92)	(-17.14)	(-11.02)	(4.65)	(5.47)	(11.55)	(2.99)	(3.21)	
**ITH**	-0.5741^***^	-0.0004	-0.0102^***^	0.1206^***^	-5.8432^***^	1.0958^***^	1.6214^***^	-8.9734^***^	Controlled
	(-5.34)	(-1.43)	(-2.84)	(2.93)	(-3.03)	(22.18)	(6.16)	(-10.96)	
**CAG**	-0.3248^**^	-0.0011^**^	0.0185^***^	-0.1266	-7.4566^***^	0.7252^***^	-1.2572^***^	0.6696	Controlled
	(-2.25)	(-2.17)	(4.83)	(-3.27)	(-2.87)	(7.34)	(-7.51)	(0.72)	
**RET**	21.7598^***^	-0.0176^**^	0.0075^**^	-0.0345	-44.0765^***^	0.3774^***^	0.4326^***^	1.2426^*^	Controlled
	(15.96)	(-3.36)	(2.30)	(-1.02)	(-21.78)	(7.43)	(13.10)	(1.73)	
**CTR**	-2.0452^***^	-0.0123^***^	0.0093	-0.0224	17.4815^***^	0.1915	-0.2286	1.0110	Controlled
	(-8.74)	(-3.34)	(1.09)	(-0.24)	(4.13)	(1.23)	(-0.97)	(1.01)	
**MEA**	-1.7265^***^	-0.0035^***^	-0.0067^**^	0.0539^*^	20.7619^***^	0.4964^***^	-0.0562^*^	0.2846	Controlled
	(-16.12)	(-9.24)	(-2.53)	(1.95)	(10.77)	(14.94)	(-1.67)	(0.87)	
**CMA**	0.8996^***^	-0.0018^***^	-0.0021	-0.0439^*^	-24.1221^***^	-0.0078	2.3086	24.8012^***^	Controlled
	(11.91)	(-3.49)	(-0.85)	(-1.67)	(-16.75)	(-0.15)	(1.42)	(6.78)	
**ACP**	1.6296^***^	-0.0021^*^	-0.0293^***^	0.2369^***^	-31.8844^***^	1.1152^***^	12.8004^***^	-26.3774^***^	Controlled
	(7.58)	(-1.97)	(-4.60)	(3.37)	(-9.09)	(16.21)	(10.53)	(-13.32)	
**DFI**	-0.9580^***^	-0.0075^***^	-0.0272^***^	0.2665^***^	-1.5495	-0.5471^***^	-7.3234^***^	-3.5235^**^	Controlled
	(-7.41)	(-4.72)	(-6.94)	(6.43)	(-0.63)	(-7.95)	(-15.43)	(-2.25)	
**PBT**	-0.0521	-0.0001	-0.0047	-0.1770^***^	-14.6253^***^	0.7327^***^	0.2619^***^	-0.8120	Controlled
	(-0.39)	(-0.21)	(-1.32)	(-4.83)	(-6.27)	(18.85)	(9.71)	(-1.40)	
**CBI**	0.2217	-0.0007	0.0112^***^	-0.1933^***^	-20.5418^***^	-0.4899^***^	0.1409^**^	1.5537^*^	Controlled
	(1.34)	(-1.42)	(2.99)	(-5.07)	(-7.79)	(-12.37)	(2.05)	(1.73)	
**INS**	0.1322	-0.0120^***^	0.0008	0.1442	-13.6261^***^	0.2181^***^	0.5085^**^	6.3186^***^	Controlled
	(1.08)	(-7.62)	(0.20)	(2.87)	(-6.14)	(2.92)	(2.08)	(5.72)	
**ENG**	1.1792^***^	-0.0118^***^	-0.0350^***^	0.3006^***^	-29.1662^***^	-0.6475^***^	-13.3090^***^	23.7214^***^	Controlled
	(9.32)	(-14.45)	(-9.54)	(8.38)	(-13.66)	(-11.20)	(-9.99)	(7.05)	

Note: The estimation results of common factors are presented in Table in S2 Table. The figures in brackets are t statistics.

* represents significance at the 10% level.

** represents significance at the 5% level.

*** represents significance at the 1% level.

The number of industries whose corresponding node strength S has a significant negative (positive) effect on its systemic risk contribution is 7 (7), which indicates that the effect of node strength on the system risk contribution in the industry network is not consistent. The number of industries whose betweenness centrality B has a significant negative (positive) effect on its systemic risk contribution is 13 (1), suggesting that the larger an industry’s betweenness centrality in the network is, the smaller the *ΔCoVaR*, and the larger its systemic risk contribution. The number of industries whose closeness centrality F has a significant negative (positive) effect on its systemic risk contribution is 10 (3), suggesting that the larger the node’s closeness centrality in the network is, the smaller the *ΔCoVaR* and the larger its systemic risk contribution is. The number of industries whose clustering coefficient C has a significant negative (positive) effect on its systemic risk contribution is 12 (4), indicating that the larger the node’s clustering coefficient in the network is, the larger its systemic risk contribution. The number of industries whose occupation layer in the stock industry network has a significant negative (positive) effect on its systemic risk contribution is 4 (10), which means that the larger the node’s occupation layer in the network is, the larger the *ΔCoVaR*, and the smaller its systemic risk contribution.

If a node’s betweenness centrality is high, it will be more central to the network, considering its intermediary influence in the network. Thus, the corresponding industry has a larger systemic risk contribution. Generally, a larger closeness centrality is accompanied by a greater linkage effect on other industries in the network, making it is easier to spread information between them. Such industries have a greater impact on other industries and have larger systemic risk contributions. A larger occupancy layer represents a greater distance from the center industry index node and a weaker correlation between them. Thus, the corresponding industry has a larger systemic risk contribution. The node clustering coefficient of the industry reflects the edge density of its local network and the return correlation strength among industries in the local network. A large density and relevant intensity of local networks leads to greater systemic risk contribution.

### Further analysis

We verify the robustness of results by dividing the full time domain into two sub-periods of similar length: 2007–2011 and 2012–2016. The panel data regression results of the two sub-periods are shown in Table 5. The sub-period results are similar to the overall sample regression results, and there are no significant changes.

**Table 5:** Regression results for two sub-periods.

	S	B	F	l	C	lnAsset
**2007–2011**	8(6)	6(3)	10(4)	3(11)	12(4)	0(12)
**2012–2016**	4(10)	9(1)	10(3)	4(5)	15(1)	12(2)
	**leverage**	**ROA**	**Gdpgr**	**CPI**	**r**	**ex**
**2007–2011**	6(7)	8(5)	2(14)	12(3)	6(4)	12(3)
**2012–2016**	7(5)	13(3)	4(7)	3(12)	0(12)	0(16)

Note: The numbers inside (outside) the parentheses represent the number of sectors in which the variable coefficient has a significantly negative (positive) effect.

**Fig 5:**
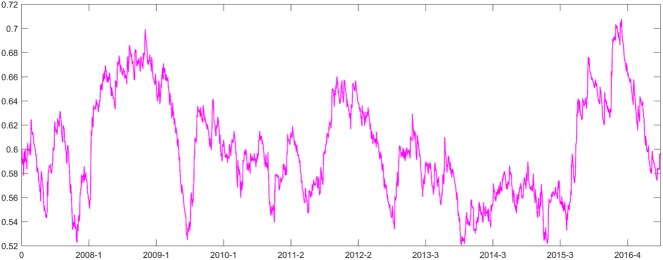
The dynamic threshold of the CSI 300 two-tier industry index network.

Moreover, we use different network construction methods to verify the robustness of the effect of network topology on systemic risk contribution. Both PMFG and MST are strongly related to single-linkage cluster analysis, and they can account for the heterogeneity of similarities, or influences, that are typically present at different correlation scales in complex systems. PMFG method is similar to MST method, but it preserves more of the information in the network. The network constructed using PMFG is much larger and more complicated with respect to the number of edges and topology than the network constructed using MST, which makes the extraction and analysis of effective information more difficult and complicated. However, the MST and threshold models rely on rather complementary concepts, and their properties can shed light on different aspects of a system. A threshold network is a network in which the correlation influence values exceed a given threshold. MST networks are based on hierarchical clustering and enable one to consider the heterogeneity of interactions by maintaining hierarchical information, in order to retain information about elements of poorly interacting groups, which could not be selected using a threshold method [44]. Hence, the threshold method is used to conduct the robustness test. We introduce a dynamic threshold $Q_{d}(t)=\frac{2}{N(N\text{-}1)}\sum_{i=1}^{N}\sum_{j=i+1}^{N}\rho_{ij}$, and it’s changes are shown in Fig 5. *Q*_*d*_*(t)* takes the average over all stocks in a single time step, and fluctuates synchronously with the cross-correlations *ρ*_*ij*_*(t)* [57]. Therefore, the dynamic threshold may suppress the large fluctuations induced by *ρ*_*ij*_*(t)* to create a stable network structure. We consider the dynamic threshold of *θ* = *Q*_*d*_*(t)*. The dynamic network is filtered using the dynamic threshold method, and the network topology indicators are calculated.

**Fig 6:**
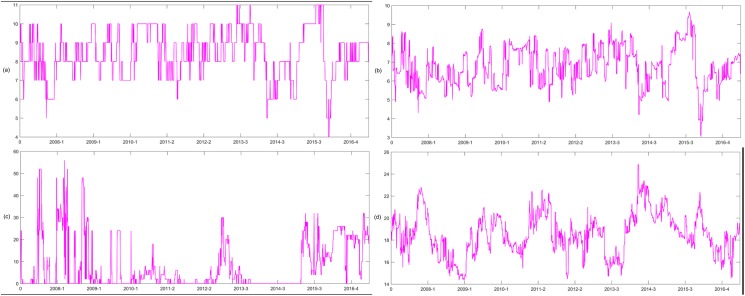
ENG’s dynamic evolution of node degree (a), node strength (b), betweenness centrality(c), and closeness centrality (d) in the threshold network.

Since the network formed by the threshold method is a non-connected network, the node occupancy layer cannot be calculated. We calculate the topological indicators mentioned above in addition to the node occupancy layer, and the evolution of the topology structure of the ENG is shown in Fig 6. The effect of network topology on the systemic risk contribution is analyzed by using the SUR panel data model, and the regression results are shown in Table 6. The numbers of industries whose corresponding node strength, betweenness centrality, closeness centrality and clustering coefficient in the financial network have significant negative (positive) effects on its systemic risk contribution are 7 (8), 9 (2), 10 (4) and 11 (5), respectively. The results do not significantly differ from the results of the MST method, so the main conclusions of this paper are robust.

**Table 6:** Panel data model estimation results.

Industry	S	B	F	C	lnAsset	leverage	ROA	Common factors
**CRE**	1.1115^***^	-0.0063^***^	0.0079	-30.9981^***^	0.6768^***^	-1.2886^***^	-0.9692^***^	Controlled
	(16.28)	(-9.52)	(1.26)	(-25.36)	(12.26)	(-18.45)	(-3.28)	
**FBA**	-1.2005^***^	-0.0031^**^	-0.0949^***^	11.6106^***^	0.1996	0.7784	-27.6846^***^	Controlled
	(-4.31)	(-2.15)	(-4.10)	(-2.70)	(-0.61)	(-0.79)	(-15.53)	
**DAP**	1.0705^***^	0.0004	-0.1262^***^	-37.2004^***^	0.1723^***^	-0.3330	-10.6226^***^	Controlled
	(8.63)	(0.75)	(-9.79)	(-19.33)	(4.89)	(-1.06)	(-9.70)	
**ITF**	0.5106^***^	-0.0116^***^	-0.0990^***^	-26.6604^***^	0.3878^***^	-2.9514^***^	-12.9331^***^	Controlled
	(3.10)	(-8.65)	(-8.40)	(-10.29)	(3.40)	(-9.93)	(-14.93)	
**UTL**	-2.1301^***^	-0.0747^***^	-0.0353^***^	21.2333^***^	0.7512^***^	1.2504^***^	1.3065^*^	Controlled
	(-13.65)	(-24.12)	(-2.81)	(8.85)	(15.58)	(4.91)	(1.87)	
**ITH**	-0.6968^***^	-0.0021	-0.0251^***^	-2.9565	1.1472^***^	1.0821^***^	-10.5853^***^	Controlled
	(-5.57)	(-1.60)	(2.38)	(-1.47)	(23.28)	(4.01)	(-13.00)	
**CAG**	-1.3341	-0.0028	-0.0786^**^	5.2955^**^	0.7053^***^	-1.1254^***^	2.2729^*^	Controlled
	(-8.55)	(0.91)	(-7.66)	(2.05)	(7.43)	(-7.09)	(2.45)	
**RET**	1.7474^***^	-0.0160	0.0000	-44.5848^***^	0.3422^***^	0.2548^*^	1.4105^*^	Controlled
	(15.69)	(-1.12)	(0.01)	(-22.01)	(6.84)	(1.83)	(1.92)	
**CTR**	-2.6480^***^	-0.0042	0.0086^**^	26.8631^***^	0.0787	-0.0378	-0.2469	Controlled
	(-10.57)	(0.14)	(2.34)	(6.06)	(0.49)	(-0.15)	(0.25)	
**MEA**	-2.2292^***^	-0.0027^***^	-0.0118^**^	28.7194^***^	0.5273^***^	0.0143	-0.9736^***^	Controlled
	(-20.48)	(-2.96)	(-3.08)	(15.36)	(15.64)	(0.42)	(-2.91)	
**CMA**	0.5866^***^	0.0033^*^	-0.0140	-19.2708^***^	0.0221	1.7952	19.1286	Controlled
	(6.08)	(1.90)	(-3.08)	(-11.81)	(0.43)	(1.21)	(5.51)	
**ACP**	1.8356^***^	-0.0117	-0.0435^***^	-35.2158^***^	1.2123^***^	3.0629^***^	-23.5085^***^	Controlled
	(9.80)	(-2.91)	(-4.20)	(-10.96)	(17.65)	(11.63)	(-11.54)	
**DFI**	-0.6822	-0.0457^***^	-0.0333^***^	-4.3910^*^	-0.3965^***^	-6.0922^***^	-6.3850	Controlled
	(-4.83)	(-3.32)	(5.60)	(-1.71)	(-5.47)	(-12.36)	(-3.91)	
**PBT**	0.0938	0.0136^***^	0.0187^*^	-15.7843^***^	0.7821^***^	0.3441^***^	-0.4256	Controlled
	(0.63)	(3.23)	(1.86)	(-6.47)	(20.03)	(12.74)	(-0.73)	
**CBI**	0.7878^**^	-0.0158^*^	0.0015	-30.3793^***^	-0.4490^***^	0.3329^***^	-0.1785	Controlled
	(5.03)	(-9.88)	(0.11)	(-11.56)	(-11.37)	(4.66)	(-0.19)	
**INS**	-0.2352	-0.0324	-0.0267^*^	-8.9017^***^	0.2535^***^	0.0422	6.1893^***^	Controlled
	(-1.28)	(0.84)	(-1.88)	(-3.14)	(3.34)	(0.17)	(5.51)	
**ENG**	0.02844^**^	-0.0119^***^	-0.0398^***^	-15.2070^***^	-0.6966^***^	-18.6896^***^	14.5459^***^	Controlled
	(2.00)	(-5.15)	(-7.32)	(-6.63)	(-11.96)	(-14.98)	(4.27)	

Note: The estimation results of common factors are presented in Table in S2 Table. The figures in brackets are t statistics.

* represents significance at the 10% level.

** represents significance at the 5% level.

*** represents significance at the 1% level.

## Conclusion

This paper investigates the effects of the network topology of a stock industry brings on the systemic risk contribution of various industries. We choose the CSI 300 index and its two-tier industry indices data, and use the dynamic time-varying t-copula and GARCH model to calculate the CoVaR and *ΔCoVaR* of the CSI 300 two-tier industry indices. We find that the systemic risk contribution of small-scale industries such as CRE, FBS, DAP, and ITF, are greater than those of large-scale industries, such as CBI, INS and ENG. The stock industry index network is constructed based on the DDCs between industry indices, and the network topology of the analysis node is observed using the MST method. Finally, SUR of the panel data is utilized to analyze the relationship between the network topology and the systemic risk contribution. Due to existence of information spillover and industry associations, changes in the local network structure of the stock industry often lead to changes in the systemic risk contribution of that industry. Industry with larger betweenness centrality, closeness centrality, and clustering coefficient and smaller node occupancy tend to be associated with larger systemic risk contributions. The more important the position of the industry in the network is, the stronger its capacity of information dissemination, the closer the industry association between it and other industries, and the stronger its risk spillover effect on other industries and the whole market.

The policy implications of this paper are reflected in the following aspects: First, there are some industry nodes with large systemic risk contribution in the Chinese stock market. As a consequence, regulators can identify specific industry nodes based on the systemic risk contribution of each industry in the stock market network. Effective supervision can decrease the potential for an industry’s risk and influence to spread in the network, in order to maintain the stability and safety of the financial system. Second, regulators can use network topological indicators such as betweenness centrality, closeness centrality, clustering coefficient, and node occupancy layer, as basic indices to measure the relevance of industry indices to more effectively assess the importance of the industry system.

## Supporting information

S1 Table Analyzed industries. (DOC)

S2 Table Panel data model estimation results of common factors. (DOC)

S1 Text Data underlying the findings. (RAR)
