# Supplementary material for: Does network topology influence systemic risk contribution? A perspective from the industry indices in Chinese stock market
Source: PLoS One. 2017 Jul 6;12(7):e0180382. doi: 10.1371/journal.pone.0180382 (PMC5500295; doi:10.1371/journal.pone.0180382)
Supplement: S1 Table — (DOC) [file pone.0180382.s001.doc]

**S1 Table. Analyzed industries**

| Node label | Code | Industry |
| --- | --- | --- |
| 1 | ENG | 300 Energy |
| 2 | CMA | 300 Materials |
| 3 | CAG | 300 Capital Goods |
| 4 | CTR | 300 Trans |
| 5 | ACP | 300 Auto and Component |
| 6 | DAP | 300 Durables&Apparel |
| 7 | MEA | 300 Media |
| 8 | RET | 300 Retail |
| 9 | FBA | 300 Food&Beverage |
| 10 | PBT | 300 Pharma&Biotech |
| 11 | CBI | 300 Banks |
| 12 | DFI | 300 Diversified financials |
| 13 | CRE | 300 Real Estate |
| 14 | ITF | 300 IT Software |
| 15 | ITH | 300 IT Hardware |
| 16 | UTL | 300 Utilities |
| 17 | INS | 300 Insurance |
