# Supplementary material for: Does network topology influence systemic risk contribution? A perspective from the industry indices in Chinese stock market
Source: PLoS One. 2017 Jul 6;12(7):e0180382. doi: 10.1371/journal.pone.0180382 (PMC5500295; doi:10.1371/journal.pone.0180382)
Supplement: S2 Table — (DOC) [file pone.0180382.s002.doc]

**S2 Table. Panel data model estimation results of common factors**

| Industry | *MST model* | | | | *Threshold model* | | | |
| --- | --- | --- | --- | --- | --- | --- | --- | --- |
|  | *GDPgr* | *CPIgr* | *R* | *Ex* | *GDPgr* | *CPIgr* | *R* | *Ex* |
| CRE | -0.0617^***^  -4.09 | 0.0174^*^  1.76 | 0.0447^**^  2.57 | 0.2525^***^  2.69 | -0.0801^***^  -5.35 | 0.0197^**^  2.02 | 0.0644^***^  3.76 | 0.6060  6.47 |
| FBA | 0.0866^***^  2.97 | 0.0527^*^  2.65 | 0.2239^***^  -6.78 | -1.6641^***^  -9.59 | 0.0666^**^  2.11 | 0.0256  1.21 | 0.3295^***^  9.31 | -1.1753^***^  -6.39 |
| DAP | -0.195  -1.18 | 0.0379  3.52 | 0.1094^***^  5.75 | -0.4014  -4.24 | -0.0320^*^  -1.78 | 0.0184  1.57 | 0.1384^***^  6.68 | -0.4802^***^  -4.77 |
| ITF | -0.0122  -0.64 | 0.0756^***^  5.79 | 0.1297^***^  5.95 | -0.2769^***^  -2.69 | -0.0245  -1.18 | 0.0568^***^  4.05 | 0.1802^***^  7.78 | -0.1677  -1.53 |
| UTL | -0.0303  -1.49 | 0.0226^*^  1.73 | 0.0227  1.12 | -0.1020  -0.97 | 0.0165  0.81 | -0.0303^**^  -2.41 | 0.0478^**^  2.18 | -0.1772^*^  -1.69 |
| ITH | 0.0621^***^  3.47 | -0.0481  -4.19 | 0.0322  1.58 | -0.2673^**^  -2.54 | -0.0493^***^  2.69 | 0.0508^***^  -4.41 | 0.0361^*^  1.78 | -0.1072  -1.00 |
| CAG | 0.0560^**^  2.27 | -0.0758^**^  -5.11 | 0.0479^***^  1.85 | -0.8647  -7.55 | 0.0522^**^  2.13 | -0.0586^***^  -3.95 | 0.0343  1.33 | -0.7051  -6.27 |
| RET | 0.0861^***^  4.51 | -0.0252^**^  -2.09 | 0.0319  1.56 | 0.5281***  -5.12 | 0.0699^***^  3.66 | -0.0164  -1.39 | 0.0197  0.96 | -0.5322^***^  -5.19 |
| CTR | -0.0299  -0.82 | 0.1855^***^  7.89 | 0.1326^***^  -3.17 | 0.7632^***^  -3.79 | -0.0331  -0.90 | 0.1948^***^  8.34 | -0.1496^***^  -3.61 | 0.8155^***^  -4.01 |
| MEA | 0.0816^***^  5.94 | -0.0109  -1.25 | 0.0760^***^  4.95 | -0.2558^***^  -3.50 | 0.0882^***^  6.19 | -0.0195^**^  -2.17 | -0.0876^***^  5.55 | -0.2260^***^  -3.05 |
| CMA | -0.0931^***^  -5.55 | -0.0628^*^  -5.93 | 0.0686^***^  3.65 | -0.0829  -0.94 | -0.0966^***^  -5.69 | -0.0609^***^  -5.63 | 0.0781***  4.15 | -0.0912  -1.02 |
| ACP | 0.1539^***^  4.89 | 0.1478^***^  6.95 | -0.1097^***^  3.17 | 0.2158  1.18 | 0.1439^***^  4.56 | 0.1470^***^  6.97 | 0.1338^***^  3.90 | 0.5167^***^  2.85 |
| DFI | 0.2494  9.71 | 0.2312^***^  -13.89 | 0.1145^***^  4.00 | -1.0875^***^  -8.24 | 0.2605  9.73 | -0.2510^***^  -14.70 | 0.1659^***^  5.62 | -1.1377^**^  -8.35 |
| PBT | 0.0402^*^  1.88 | 0.0605***  -4.59 | 0.0318  1.37 | -0.6495^***^  -6.14 | -0.0114  0.52 | -0.0711^***^  -5.26 | 0.0875^***^  3.71 | -0.4533^***^  -4.19 |
| CBI | -0.1420^*^  -5.97 | 0.0329**  2.19 | 0.0988  3.73 | -0.2303^*^  -1.89 | -0.14868^***^  -6.32 | 0.0427^***^  2.89 | 0.1065^**^  4.09 | -0.1880  -1.55 |
| INS | 0.0841^***^  -3.70 | 0.0376^***^  2.61 | 0.0613^***^  2.40 | -0.1598  -1.10 | -0.0827^***^  -3.56 | 0.0352^**^  2.41 | 0.0747^***^  2.95 | 0.0342  0.23 |
| ENG | -0.1983^***^  -8.69 | 0.0348^***^  2.44 | 0.0408^*^  1.65 | 0.0213  0.17 | -0.1647^***^  -7.25 | 0.0226  1.57 | 0.0706^***^  2.82 | 0.3147^***^  2.63 |

Note: The figures in brackets are t statistics. * represents significance at the 10% level. ** represents significance at the 5% level. *** represents significance at the 1% level.
